# Revealing drug targets for sepsis: An association study integrating Mendelian randomization, eQTL, pQTL, and protein–protein interaction networks

**DOI:** 10.1097/MD.0000000000048728

**Published:** 2026-05-22

**Authors:** Enkui Lu, Yanan Zhang, Xuebo Shao, Junnan Chen, Mengping Jiang, Weidong Tang

**Affiliations:** a The First People’s Hospital of Fuyang, Hangzhou, Zhejiang, China.

**Keywords:** B3GALT6, bioinformatics, Mendelian randomization, plasma protein, sepsis

## Abstract

This study aims to reveal drug targets for sepsis: an association study integrating Mendelian randomization (MR), expression quantitative trait loci, protein quantitative trait loci, and protein–protein interaction networks. We identified 43 druggable proteins associated with sepsis, with *B3GALT6* emerging as a key player (*P* = 5.24E−06). Sensitivity analyses indicated no significant heterogeneity among the proteins tested, reinforcing the robustness of our findings. Bioinformatics analyses demonstrated that B3GALT6 expression was significantly lower in sepsis patients compared to healthy controls across multiple datasets (*P* < .001), suggesting its potential utility as a diagnostic biomarker. Additionally, Gene Ontology (GO) and Kyoto Encyclopedia of Genes and Genomes (KEGG) enrichment analyses revealed critical pathways altered in sepsis, including multivesicular body assembly and ubiquitin-dependent protein catabolic processes. Our results highlight *B3GALT6* as a promising therapeutic target and prognostic marker for sepsis, with implications for future research aimed at developing innovative treatment strategies. Overall, this study provides a foundation for further exploration of druggable proteins in sepsis, emphasizing the need for additional investigations to validate these findings in clinical settings and facilitate the translation of these insights into effective therapeutic interventions.

## 1. Introduction

Sepsis, a life-threatening organ dysfunction syndrome triggered by a dysregulated host response to infection, remains a leading cause of global mortality. Contemporary epidemiological studies estimate its annual global incidence ranges from 276 to 678 cases per 1,00,000 population, with associated mortality rates persisting between 22.5% and 26.7% despite advances in critical care and antimicrobial therapies.^[1,2]^ The rate of hospitalization in sepsis increases year by year, especially in elderly patients and those with underlying diseases, showing its significant impact on public health.^[3]^ The COVID-19 pandemic has further complicated this epidemiological landscape, with SARS-CoV-2 infections contributing to both a surge in sepsis incidence and a paradigm shift in our understanding of sepsis pathophysiology.^[1]^ Current management primarily focuses on infection control and organ support, yet these approaches fail to address the underlying molecular mechanisms driving systemic hyperinflammation, immune paralysis, and metabolic derangements.^[4]^ The heterogeneous pathophysiology of sepsis, coupled with the paucity of targeted therapies, has led to decades of failed clinical trials, emphasizing the necessity to identify causal molecular pathways amenable to intervention.

The pathobiology of sepsis involves complex interactions between pathogen-associated molecular patterns and damage-associated molecular patterns, triggering cascades of pro-inflammatory cytokine release, endothelial dysfunction, and coagulation abnormalities.^[5,6]^ While genome-wide association studies (GWAS) have identified genetic variants associated with sepsis susceptibility and outcomes, these findings often lack mechanistic interpretation and fail to distinguish correlation from causation.^[7]^ However, translating these genetic insights into therapeutic targets remains challenging. While observational studies have identified numerous biomarkers (such as IL-6, TNF-α) and candidate pathways their causal roles in sepsis pathogenesis remain unproven.^[8]^ Confounding factors such as reverse causation, environmental influences, and comorbidities complicate traditional epidemiological approaches, necessitating methodologies robust to these biases.

Mendelian randomization (MR), which leverages genetic variants as instrumental variables to infer causality between exposures and outcomes, has emerged as a powerful tool to prioritize therapeutic targets.^[9]^ By mimicking randomized controlled trials through genetic proxies, MR minimizes confounding and establishes temporality, thereby addressing limitations of conventional observational studies.^[10]^ Recent innovations integrating MR with expression quantitative trait loci (eQTL) and protein quantitative trait loci (pQTL) data enable systematic prioritization of drug targets by linking genetic associations to gene expression and protein abundance.^[11]^ Furthermore, bioinformatics technology can elucidate functional pathways connecting putative targets to sepsis pathogenesis, enhancing biological plausibility. Validating MR-derived targets can pinpoint hub molecules controlling disease-related pathways and enhance the biological plausibility of candidate targets. Despite these advances, no study has comprehensively applied this integrative framework to sepsis, leaving critical knowledge gaps in understanding causal mediators and their interplay.

Recent work pioneered the application of MR to sepsis drug target discovery, identifying PSMA4, IFNAR2, and LY9 through integration of 2 sepsis GWAS datasets (IEU-B-4980 and IEU-B-69) and blood cis-eQTL data. This study laid a critical foundation for genetic-driven target identification in sepsis, particularly highlighting PSMA4 as a promising candidate via robust colocalization analysis.^[12]^ Nevertheless, key limitations persist: reliance solely on eQTL, lack of functional characterization, and no evaluation of diagnostic potential for clinical translation. To build on this foundational work, the present study advances the field with a multidimensional integrative framework: we combine MR with both eQTL and pQTL data to strengthen causal inference between genetics, protein expression, and sepsis risk – an approach not previously applied in sepsis research.

In this study, we present a comprehensive association framework integrating MR, eQTL/pQTL colocalization, and bioinformatics technology to identify and validate drug targets for sepsis. Our analysis systematically investigates causal relationships between genetically predicted plasma proteins, gene expression, and sepsis risk, while elucidating target druggability and pathway centrality. This multi-dimensional strategy aims to overcome the limitations of reductionist approaches, offering actionable insights for therapeutic development against this heterogeneous syndrome.

## 2. Materials and methods

### 2.1. GWAS data source

#### 2.1.1. GWAS summary statistics of pQTL

Cis-protein quantitative trait loci (cis-pQTLs) specifically refer to the pQTL located near its target gene or protein genome (at cis position). In this study, we used 2 sources of pQTL. To screen for druggable viable proteins for subsequent analysis, we used the cis-pQTL data from the study, including 738 cis-SNP for 734 proteins conducted by Zheng et al.^[13]^ The GWAS summary statistics for plasma proteins originate from data released by Ferkingstad et al.^[14]^ This study measured 4907 plasma proteins in 35,559 subjects. The following criteria were used to select the instrumental variables: showed genome-wide significant association (*P* < 1 × 10^−5^); were located outside the major histocompatibility complex region (chr6, 26–34 Mb); evaluated the independence of the selected SNPs using pairwise linkage disequilibrium (LD) to exclude those in LD (*R*^2^ > 0.001, window distance < 10,000 kb); the *F*-statistic was computed for each SNP, and the strength of each instrumental variable (IV) was evaluated based on the variance in each exposure explained by the SNP, calculated as


$$\begin{array}{l} F=\frac{\left(N-K-1\right)/K}{R^{2}/\left(1-R^{2}\right)}, \end{array}$$

with *K* representing the count of genetic variants and N the sample size. SNPs exhibiting an *F*-statistic lower than 10 were discarded.

#### 2.1.2. GWAS summary statistics of eQTL

The eQTL dataset was sourced from eQTLGen (https://eqtlgen.org/).^[15]^ In this study, a GWAS was performed on 30,935 European individuals, with approximately 20,000 SNPs analyzed. Instrumental variables were defined as SNPs showing genome-wide significant associations (*P* < 1 × 10^−8^).

#### 2.1.3. GWAS summary statistics of sepsis

The GWAS summary statistics for sepsis were obtained from data released by the FinnGen study.^[16]^ The study performed a GWAS on 4,14,305 European individuals (N_case_ = 15,156, N_control _= 3,99,149), with approximately 21 million SNPs analyzed.

### 2.2. MR analysis

A 2-sample bidirectional MR analysis was performed to evaluate the reciprocal causal relationships between plasma proteins and sepsis, representing the total effect. The inverse-variance weighted (IVW) method under a random-effects model served as the primary statistical approach.^[17]^ Four additional methods – MR-Egger,^[18]^ weighted median,^[19]^ weighted mode,^[19]^ and simple mode – were used to confirm the reliability of the IVW results. The IVW method requires that all SNPs meet the 3 Mendelian randomization assumptions. The MR-Egger intercept provides a test for horizontal pleiotropy, and the weighted median and weighted mode methods were used to assess the presence of a causal relationship.

### 2.3. Sensitivity analysis

Assessment of SNP heterogeneity was performed using Cochran *Q* statistic and funnel plots.^[20]^ Horizontal pleiotropy was detected using the MR-Egger intercept method^[17]^ and the “MR-PRESSO” approach.^[21]^ If outliers were detected, they were removed, and the MR causal estimates were reassessed. If heterogeneity remained high after outlier removal, the stability of the results was evaluated using a random-effects model, which is less susceptible to weaker SNP-exposure associations. As a final step, a leave-one-out analysis was carried out to determine the contribution of each SNP to the overall causal estimates.

### 2.4. Bayesian co-localization analysis

To assess whether the 2 traits share the same causal variant, we used the probability of the causal variable implemented in the “coloc” package with default parameters. Bayesian co-localization provides posterior probabilities for 5 hypotheses concerning whether a single variant is shared between 2 traits.^[22]^ In this analysis, we focused on the posterior probability of hypothesis 3 (PPH3), which posits that eQTL and sepsis are associated with the region via distinct variants, and the posterior probability of hypothesis 4 (PPH4), which posits that both traits are associated with the region via a shared variant. Using coloc.abf, a gene was considered to have evidence of co-localization if the gene-based PPH4 exceeded 80%, according to at least 1 algorithm.^[23,24]^

### 2.5. Bioinformatics analysis

RNA-seq data and corresponding clinical information of sepsis patients were obtained from the GEO database.^[25-27]^ The ROC analysis was performed to assess the effect of the genes on the patients. To identify differentially expressed genes (DEGs), differential analysis was conducted using the “limma” package. Genes with significant differences in expression levels were selected based on an *P* value < .05. Additionally, we utilized the Human Protein Atlas database^[28]^ to investigate the protein distribution across various tissues and immune cells.

### 2.6. GO and KEGG enrichment analysis

The functions of up-regulated and down-regulated DEGs were examined. Gene Ontology (GO) enrichment analysis was employed to assess the biological pathways associated with the DEGs, with a focus on biological processes (BP), molecular functions (MF), and cellular components (CC). The BP, CC, and MF of TOP10 were visualized according to the pvalue ranking. Kyoto Encyclopedia of Genes and Genomes (KEGG) enrichment analysis was also performed using the “ggplot2” package in R software.

### 2.7. The protein–protein interaction (PPI) network

Protein–protein interaction network consists of individual proteins that interact with each other. The STRING database was used to search for interactions between known and predicted proteins.^[24]^ In this study, the PPI network was constructed using the STRING database, with a minimum correlation coefficient >0.400 as a standard. Set the maximum number of interactors to be displayed to first shell no more than 50 interactors.

### 2.8. Statistical analysis

Mendelian randomization analyses were conducted using the TwoSampleMR package (version 0.5.6) in R (version 4.2.1). MR-PRESSO analysis was performed with the MR-PRESSO package. The phenoscanner V2 package was taken to remove confounders. The web-based mRnd tools was employed to evaluate the statistical power for assessing the causal power between exposure and outcome.^[29]^

## 3. Results

### 3.1. MR analysis of druggable available proteins

Two-sample MR analysis identified 43 druggable proteins associated with sepsis, with the top associations observed for CRP, SIGLEC9, DUSP13, SCARF1, and OSMR (Table 1).

**Table 1:** Mendelian randomization causal effect estimates of the druggable proteins on the onset of sepsis from the study by Zheng et al.

Outcome	Exposure	Method	n_snp_	*b*	SE	*P* value	OR (95% CI)
CRP	Sepsis	Wald ratio	1	0.342	0.083	3.85E−05	0.505 (1.408−1.196)
SIGLEC9	Sepsis	Wald ratio	1	−0.032	0.010	1.28E−03	−0.012 (0.969−0.950)
DUSP13	Sepsis	Wald ratio	1	−0.124	0.040	1.66E−03	−0.047 (0.883−0.817)
SCARF1	Sepsis	Wald ratio	1	−0.079	0.026	2.01E−03	−0.029 (0.924−0.879)
OSMR	Sepsis	Wald ratio	1	−0.198	0.066	2.78E−03	−0.068 (0.820−0.720)
SMPD1	Sepsis	Wald ratio	1	0.139	0.047	3.20E−03	0.231 (1.149−1.048)
NOV	Sepsis	Wald ratio	1	−0.223	0.076	3.28E−03	−0.074 (0.800−0.689)
B3GALT6	Sepsis	Wald ratio	1	−0.214	0.073	3.31E−03	−0.071 (0.808−0.700)
SPARCL1	Sepsis	Wald ratio	2	0.055	0.019	4.63E−03	0.092 (1.056−1.017)
ASIP	Sepsis	Wald ratio	1	−0.076	0.028	6.25E−03	−0.022 (0.927−0.878)
IGF2R	Sepsis	Wald ratio	1	−0.055	0.022	1.49E−02	−0.011 (0.947−0.906)
B3GNT8	Sepsis	Wald ratio	1	−0.040	0.017	1.58E−02	−0.008 (0.961−0.930)
SPINK1	Sepsis	Wald ratio	1	0.135	0.057	1.72E−02	0.247 (1.145−1.024)
ERAP2	Sepsis	Wald ratio	1	−0.027	0.011	1.73E−02	−0.005 (0.973−0.952)
MANEA	Sepsis	Wald ratio	1	0.035	0.015	1.83E−02	0.064 (1.035−1.006)
BPI	Sepsis	Wald ratio	1	−0.067	0.028	1.88E−02	−0.011 (0.936−0.885)
IL17RA	Sepsis	Wald ratio	1	0.037	0.016	1.99E−02	0.068 (1.038−1.006)
TEK	Sepsis	Wald ratio	1	−0.184	0.079	2.05E−02	−0.028 (0.832−0.712)
ADAM23	Sepsis	Wald ratio	1	0.065	0.028	2.12E−02	0.121 (1.067−1.010)
PLG	Sepsis	Wald ratio	1	−0.070	0.031	2.33E−02	−0.010 (0.933−0.878)
FAH	Sepsis	Wald ratio	1	−0.044	0.020	2.63E−02	−0.005 (0.957−0.921)
GPC5	Sepsis	Wald ratio	2	−0.032	0.015	2.80E−02	−0.003 (0.969−0.941)
KDR	Sepsis	Wald ratio	1	0.061	0.028	3.01E−02	0.116 (1.063−1.006)
NRP1	Sepsis	Wald ratio	1	−0.095	0.044	3.10E−02	−0.009 (0.909−0.834)
NTM	Sepsis	Wald ratio	1	−0.069	0.032	3.15E−02	−0.006 (0.933−0.876)
MTHFS	Sepsis	Wald ratio	1	−0.134	0.063	3.20E−02	−0.012 (0.874−0.774)
FGF2	Sepsis	Wald ratio	1	0.051	0.024	3.25E−02	0.097 (1.052−1.004)
AMY1A	Sepsis	Wald ratio	1	−0.050	0.024	3.32E−02	−0.004 (0.951−0.908)
ERLEC1	Sepsis	Wald ratio	1	0.143	0.067	3.33E−02	0.274 (1.154−1.011)
MMP12	Sepsis	Wald ratio	1	0.042	0.020	3.40E−02	0.081 (1.043−1.003)
HS6ST1	Sepsis	Wald ratio	1	−0.116	0.056	3.90E−02	−0.006 (0.890−0.797)
CRYZ	Sepsis	Wald ratio	1	0.025	0.012	4.08E−02	0.049 (1.025−1.001)
OAF	Sepsis	Wald ratio	1	0.067	0.033	4.11E−02	0.132 (1.070−1.003)
RNASE4	Sepsis	Wald ratio	1	−0.063	0.031	4.17E−02	−0.002 (0.939−0.884)
NT5C	Sepsis	Wald ratio	1	0.113	0.055	4.19E−02	0.221 (1.119−1.004)
FSTL1	Sepsis	Wald ratio	1	−0.115	0.057	4.24E−02	−0.004 (0.891−0.797)
FAM171B	Sepsis	Wald ratio	1	0.083	0.041	4.30E−02	0.164 (1.087−1.003)
CDH11	Sepsis	Wald ratio	1	−0.037	0.018	4.37E−02	−0.001 (0.964−0.930)
TIMP4	Sepsis	Wald ratio	1	−0.067	0.033	4.37E−02	−0.002 (0.936−0.877)
CNTN1	Sepsis	Wald ratio	1	0.116	0.057	4.44E−02	0.228 (1.123−1.003)
AFP	Sepsis	Wald ratio	1	−0.154	0.077	4.62E−02	−0.003 (0.857−0.737)
GSTO1	Sepsis	Wald ratio	1	0.027	0.014	4.75E−02	0.054 (1.028−1.000)
RGMA	Sepsis	Wald ratio	1	0.117	0.059	4.97E−02	0.233 (1.124−1.000)

CI = confidence interval, OR = odds ratio, SE = standard error.

**Figure 1.**
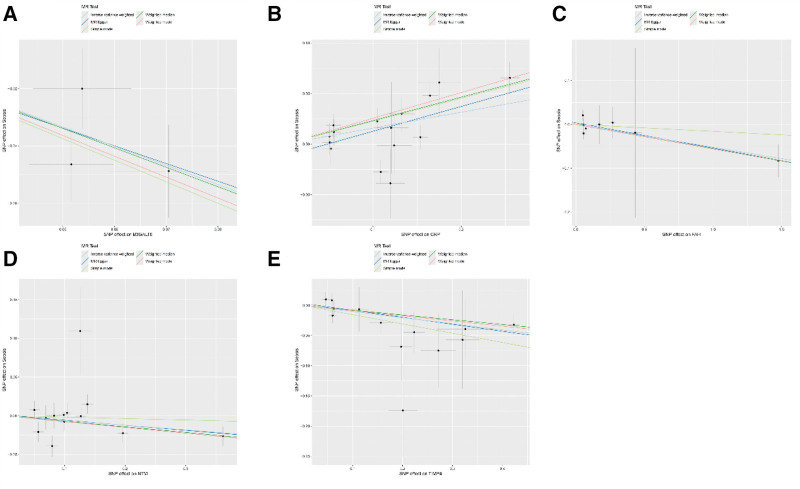
Scatter plots of the different MR model effect estimates for the rest of these 5 proteins and sepsis. (A) SNP effect on B3GALT6, (B) SNP effect on CRP, (C) SNP effect on FAH, (D) SNP effect on NTM, (E) SNP effect on TIMP4. MR = Mendelian randomization.

Further validation using pQTL data confirmed causal relationships for 5 proteins: *CRP* showed a positive association with sepsis (odds ratio [OR] = 1.171, 95% confidence interval [CI]: 1.070−1.281, *P* = 6.16E−04), while *B3GALT6* (OR = 0.513, 95% CI: 0.385−0.684, *P* = 5.24E−06), *FAH* (OR = 0.949, *P* = 3.10E−02), *NTM* (OR = 0.941, *P* = 4.72E−02), and *TIMP4* (OR = 0.904, *P* = 1.47E−03) exhibited significant protective effects (Table 2). Effect estimates from multiple MR models (IVW, MR-Egger, weighted median) were consistent, confirming robustness (Fig. 1A–E).

**Table 2:** Mendelian randomization causal effect estimates of the proteins on the onset of sepsis from the study by Ferkingstad et al.

Exposure	Outcome	Method	n_snp_	*b*	SE	*P* value	OR (95% CI)
B3GALT6	Sepsis	MR Egger	3	−0.616	1.513	7.54E−01	0.540 (0.028−10.486)
B3GALT6	Sepsis	Weighted median	3	−0.678	0.198	6.02E−04	0.508 (0.345−0.748)
B3GALT6	Sepsis	Inverse variance weighted	3	−0.668	0.147	5.24E−06	0.513 (0.385−0.684)
B3GALT6	Sepsis	Simple mode	3	−0.750	0.245	9.24E−02	0.472 (0.292−0.764)
B3GALT6	Sepsis	Weighted mode	3	−0.727	0.249	9.98E−02	0.483 (0.297−0.787)
CRP	Sepsis	MR Egger	15	0.246	0.096	2.35E−02	1.279 (1.060−1.544)
CRP	Sepsis	Weighted median	15	0.232	0.048	1.55E−06	1.261 (1.147−1.387)
CRP	Sepsis	Inverse variance weighted	15	0.158	0.046	6.16E−04	1.171 (1.070−1.281)
CRP	Sepsis	Simple mode	15	0.226	0.084	1.79E−02	1.254 (1.063−1.480)
CRP	Sepsis	Weighted mode	15	0.255	0.049	1.45E−04	1.290 (1.171−1.422)
FAH	Sepsis	MR Egger	8	−0.057	0.026	7.33E−02	0.944 (0.896−0.995)
FAH	Sepsis	Weighted median	8	−0.056	0.024	2.31E−02	0.946 (0.902−0.992)
FAH	Sepsis	Inverse variance weighted	8	−0.052	0.024	3.10E−02	0.949 (0.906−0.995)
FAH	Sepsis	Simple mode	8	−0.016	0.084	8.57E−01	0.984 (0.834−1.162)
FAH	Sepsis	Weighted mode	8	−0.055	0.025	6.43E−02	0.946 (0.901−0.994)
NTM	Sepsis	MR Egger	13	−0.066	0.057	2.77E−01	0.937 (0.837−1.048)
NTM	Sepsis	Weighted median	13	−0.072	0.030	1.82E−02	0.931 (0.877−0.988)
NTM	Sepsis	Inverse variance weighted	13	−0.061	0.031	4.72E−02	0.941 (0.885−0.999)
NTM	Sepsis	Simple mode	13	−0.018	0.054	7.40E−01	0.982 (0.883−1.092)
NTM	Sepsis	Weighted mode	13	−0.075	0.030	2.55E−02	0.927 (0.875−0.983)
TIMP4	Sepsis	MR Egger	13	−0.115	0.045	2.60E−02	0.891 (0.816−0.973)
TIMP4	Sepsis	Weighted median	13	−0.079	0.036	2.82E−02	0.924 (0.861−0.992)
TIMP4	Sepsis	Inverse variance weighted	13	−0.101	0.032	1.47E−03	0.904 (0.849−0.962)
TIMP4	Sepsis	Simple mode	13	−0.154	0.086	9.95E−02	0.858 (0.724−1.015)
TIMP4	Sepsis	Weighted mode	13	−0.086	0.036	3.58E−02	0.917 (0.854−0.985)

CI = confidence interval, OR = odds ratio.

### 3.2. Sensitivity analysis of proteins and sepsis

**Figure 2.**
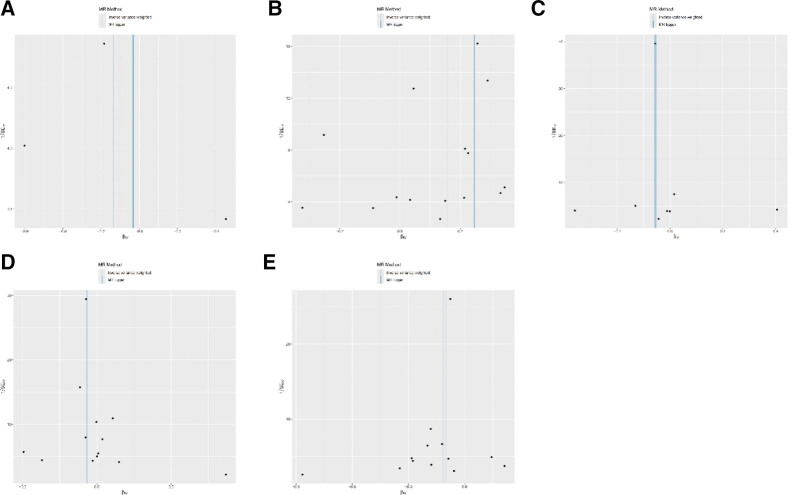
The funnel plot of 5 proteins. (A) B3GALT6, (B) CRP, (C) FAH, (D) NTM, (E) TIMP4. MR = Mendelian randomization.

**Figure 3.**
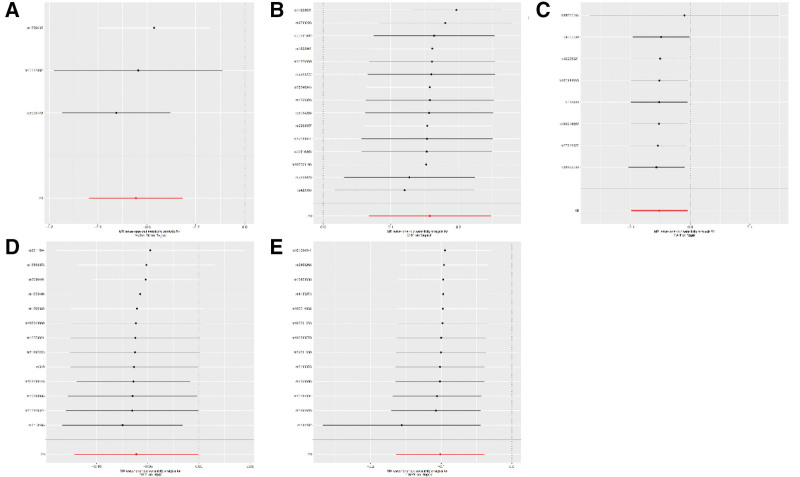
Sensitivity analysis of proteins and sepsis. (A) MR leave-one-out sensitivity analysis for test B3GALT6, (B) MR leave-one-out sensitivity analysis for test CRP, (C) MR leave-one-out sensitivity analysis for test FAH, (D) MR leave-one-out sensitivity analysis for test NTM, (E) MR leave-one-out sensitivity analysis for test TIMP4. MR = Mendelian randomization.

Cochran *Q* test revealed no significant heterogeneity for *B3GALT6, FAH, NTM*, and *TIMP4* (QP > 0.05), with only slight heterogeneity for CRP (QP < 0.05; Table 3). Funnel plots showed symmetric distribution of instrumental variables, and MR-Egger intercept tests (*P* > .05) ruled out horizontal pleiotropy (Table 4). Leave-one-out analysis confirmed that no single SNP biased the overall results, underscoring the stability of the causal inferences (Fig. 2A–E). All of the 10 proteins were measured (Fig. 3A–E) by using the leave-one-out method. All results closely β values with 0, indicating a negligible deviation from the null hypothesis. The high degree of overlap between results suggests that excluding any single SNP did not significantly change the overall results and the results were robust.

**Table 3:** Heterogeneity tests of instrument effects between plasma proteins and sepsis.

Exposure	Outcome	Method	*Q*	*Q*_df	*Q*_*P*val
B3GALT6	Sepsis	MR Egger	2.110	1	0.146
B3GALT6	Sepsis	Inverse variance weighted	2.113	2	0.348
CRP	Sepsis	MR Egger	26.406	13	0.015
CRP	Sepsis	Inverse variance weighted	28.643	14	0.012
FAH	Sepsis	MR Egger	5.612	6	0.468
FAH	Sepsis	Inverse variance weighted	5.853	7	0.557
NTM	Sepsis	MR Egger	18.460	11	0.072
NTM	Sepsis	Inverse variance weighted	18.474	12	0.102
TIMP4	Sepsis	MR Egger	10.162	11	0.516
TIMP4	Sepsis	Inverse variance weighted	10.357	12	0.585

**Table 4:** Pleiotropy tests of instrument effects between the proteins and sepsis.

Exposure	Outcome	Egger_intercept	SE	*P* value
B3GALT6	Sepsis	−0.003	0.087	.9789
CRP	Sepsis	−0.012	0.011	.313
FAH	Sepsis	0.003	0.007	.641
NTM	Sepsis	0.001	0.009	.930
TIMP4	Sepsis	0.003	0.007	.667

SE = standard error.

### 3.3. B3GALT6-eQTL was significantly negatively associated with sepsis

**Figure 4.**
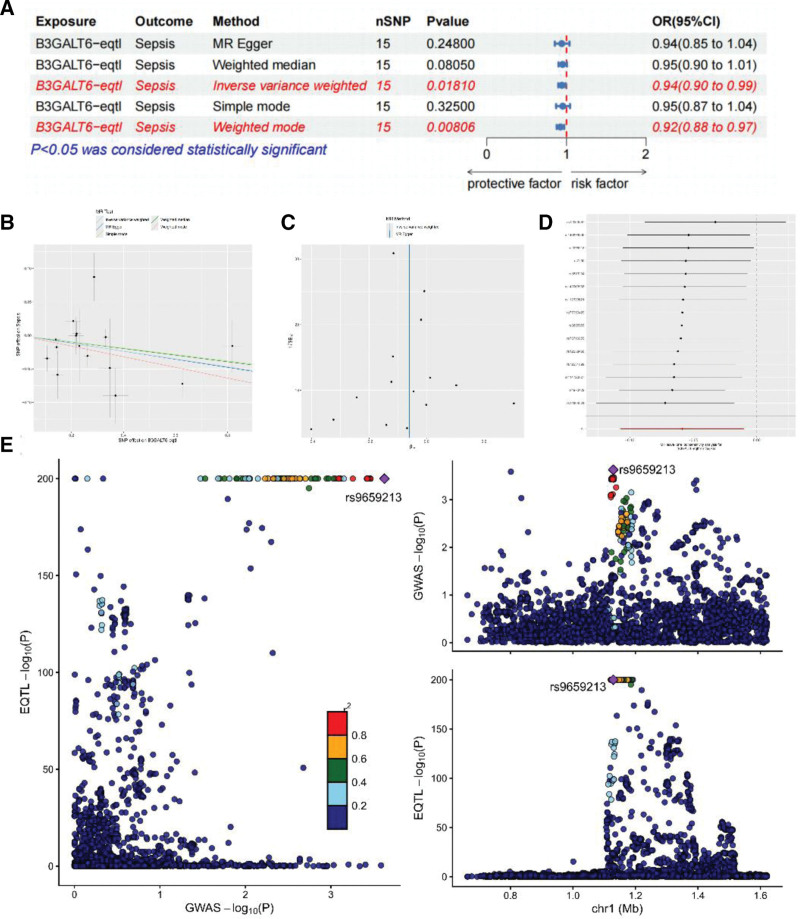
B3GALT6-eqtl showed a significant negative association with sepsis. (A) B3GALT6-eQTL and sepsis, (B) SNP effect on B3GALT6-eQTL, (C) IVW and MR-Egger regression analyses, (D) random-effects inverse-variance weighted method, (E) Bayesian co-localization analysis between B3GALT6-eqTL and sepsis. IVW = inverse-variance weighted, MR = Mendelian randomization.

MR analysis of *B3GALT6*-eQTL (15 genome-wide significant SNPs) demonstrated a negative association with sepsis (IVW: OR = 0.943, 95% CI: 0.898−0.990, *P* = .018), with consistent results across complementary models (Fig. 4A, B). No pleiotropy was detected (MR-Egger intercept *P* = .970), and Cochran *Q* test indicated no SNP heterogeneity (IVW *Q* = 26.034; MR-Egger *Q* = 26.031; Fig. 4C). Leave-one-out analysis validated result robustness (Fig. 4D), and Bayesian colocalization confirmed shared causal variants between *B3GALT6*-eQTL and sepsis (coloc.abf-PPH4 > 0.8, rs9659213; Fig. 4E). The statistical power for this association was 0.94.

### 3.4. Bioinformatics analysis of the expression of B3GALT6 in sepsis

**Figure 5.**
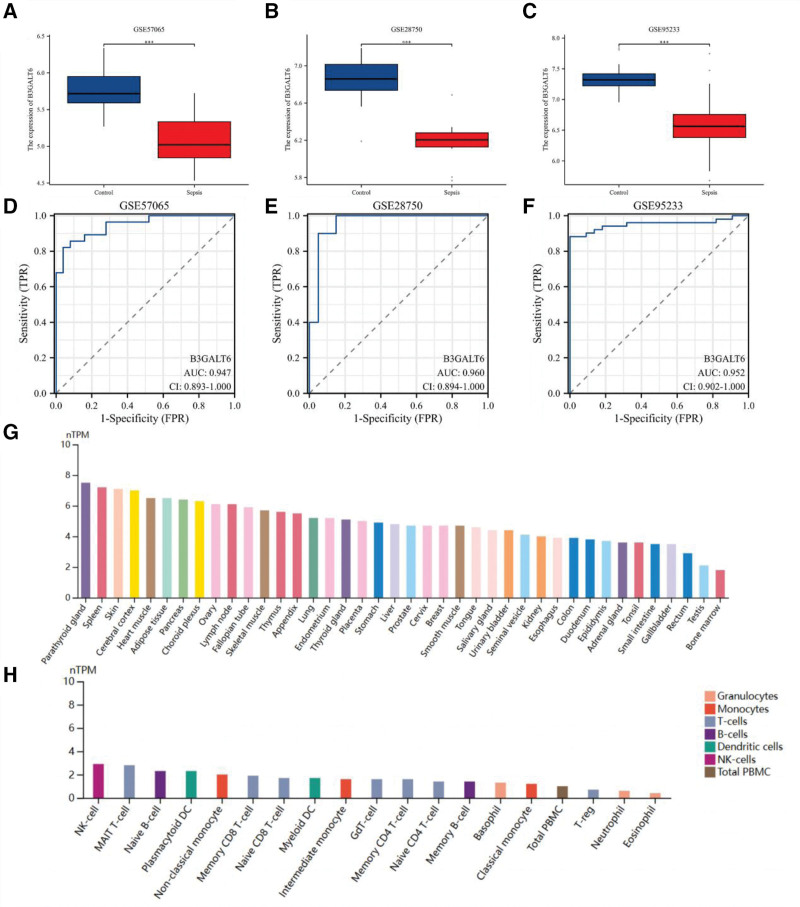
B3GALT6 expression of sepsis. (A) B3GALT6 expression in GSE57065, (B) B3GALT6 expression in GSE28750, (C) B3GALT6 expression in GSE95233 (D) ROC analysis of B3GALT6 in GSE57065, (E) ROC analysis of B3GALT6 in GSE28750, (F) ROC analysis of B3GALT6 in GSE95233, (G) B3GALT6 expressed in different organizations, (H) B3GALT6 expressed in immune cells.

*B3GALT6* expression was significantly downregulated in sepsis patients compared to healthy controls across 3 independent datasets (GSE57065, GSE28750, GSE95233; *P* < .001; Fig. 5A–C). ROC analysis demonstrated excellent diagnostic potential (AUC > 0.94 in all datasets; Fig. 5D–F). HPA database data showed *B3GALT6* expression in parathyroid gland, spleen, skin, cerebral cortex, and heart muscle, as well as in immune cells including NK cells, MAIT cells, and plasmacytoid DCs (Fig. 5G, H).

**Figure 6.**
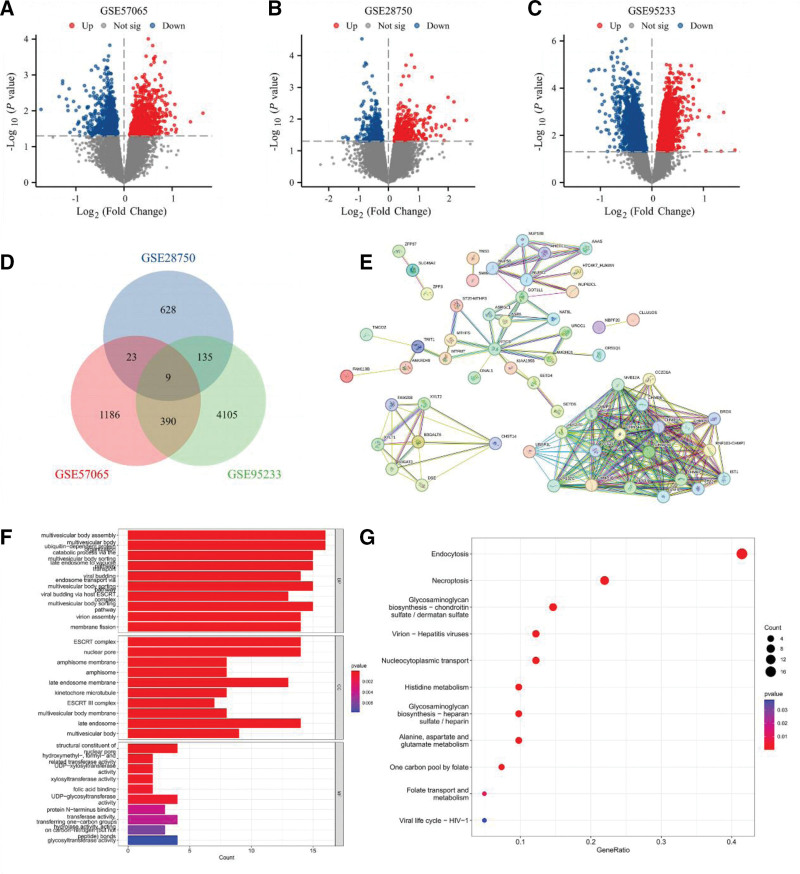
Bioinformatics analysis of the expression of B3GALT6 in sepsis. (A) Volcano plot of GSE57065, (B) volcano plot of GSE28750, (C) volcano plot of GSE95233 (D) Venn diagram, (E) PPI network of overlapping genes in Venn, (F) GO enrichment analysis, (G) KEGG enrichment analysis. GO = Gene Ontology, KEGG = Kyoto Encyclopedia of Genes and Genomes, PPI = protein–protein network.

Differential expression analysis identified 1608, 795, and 4639 DEGs in GSE57065, GSE28750, and GSE95233, respectively, with 9 shared DEGs across datasets (Fig. 6A–D). The PPI network diagram illustrates the correlation of overlapping genes (Fig. 6E). GO enrichment analysis linked these DEGs to multivesicular body assembly, ubiquitin-dependent protein catabolism, and nuclear pore function (Fig. 6F). KEGG pathways included glycosaminoglycan biosynthesis – chondroitin sulfate/dermatan sulfate, necroptosis, and endocytosis (Fig. 6G).

## 4. Discussion

Sepsis remains a global health crisis with limited targeted therapies, highlighting the need for genetically validated drug targets. Current estimates suggest that sepsis accounts for approximately 20 to 30 million cases worldwide, with around 8 million deaths each year.^[30]^ The pathophysiological complexity of sepsis, characterized by dysregulated host responses to infection, underscores the urgent need for novel therapeutic targets. Despite advancements in critical care, sepsis remains one of the most challenging conditions, with limited effective treatments beyond supportive therapies, which often leads to delayed treatment and poor outcomes.^[31,32]^ Our study integrates MR, eQTL, pQTL, and bioinformatics technology to systematically identify druggable targets for sepsis. The findings reveal critical insights into the genetic and molecular underpinnings of sepsis, highlighting potential therapeutic candidates and biological pathways, thereby enhancing our understanding of sepsis management and paving the way for future interventions.

Our MR analysis identified 43 druggable proteins associated with sepsis, with *CRP, SIGLEC9, DUSP13, SCARF1, OSMR, SMPD1, NOV, B3GALT6, SPARCL1*, and *ASIP* showing the most significant correlations. Notably, CRP demonstrated a positive causal relationship with sepsis (OR = 1.171, *P* = 6.16E−04), consistent with its established role as a biomarker of systemic inflammation.^[33]^ CRP’s positive association with sepsis aligns with its role in amplifying complement activation and cytokine release.^[34]^ However, conflicting studies report that CRP deficiency exacerbates bacterial infections, highlighting context-dependent roles.^[35]^ Our sensitivity analyses confirmed the robustness of CRP’s association, though heterogeneity (Cochran *Q P* < .05) suggests potential confounding by unmeasured pleiotropy. Furthermore, in the inflammatory response, the role of CRP is not limited to the markers, and it may play a more complex role in the regulation of inflammation. Studies have elucidated that CRP may participate in the regulatory processes underlying inflammation, positioning it as a potential therapeutic target. However, the suppression of CRP could potentially compromise vital immune defenses, necessitating a cautious approach in the development of CRP-targeted therapeutic strategies.^[36,37]^

Conversely, *B3GALT6, FAH, NTM*, and *TIMP4* exhibited significant negative correlations with sepsis, suggesting protective effects. Previous MR studies have identified inflammatory biomarkers (e.g., IL-6, TNF-α) as sepsis risk factors,^[38]^ but few have explored druggable proteins. Our findings extend prior work by integrating pQTL and eQTL data, thereby strengthening causal inference. For instance, *TIMP4*, a tissue inhibitor of metalloproteinases, showed a protective effect (OR = 0.904, *P* = 1.47E−03). The mechanism of action of *TIMP4* may be related to its inhibition of MMPs. MMPs are considered to be important factors causing endothelial barrier dysfunction and tissue damage in sepsis. *TIMP 4* was found to play a protective role by inhibiting MMPs activity and thus by reducing endothelial cell leakage and tissue degradation in sepsis.^[39]^ Similarly, FAH’s association may reflect its role in tyrosine metabolism, which modulates oxidative stress in sepsis.^[40,41]^ These may be a new direction for the study of sepsis.

*B3GALT6*, in particular, emerged as a robust candidate: its pQTL analysis confirmed a negative association with sepsis (OR = 0.943, *P* = .018), and bioinformatics validation across multiple datasets (GSE57065, GSE28750, GSE95233) revealed significantly reduced expression in sepsis patients compared to healthy controls (*P* < .001). The diagnostic potential of *B3GALT6*, evidenced by high AUC values 0.947 (95% CI: 0.893−1.000), 0.960 (95% CI: 0.894−1.000), 0.952 (95% CI: 0.902−1.000), further supports its clinical relevance. This could be a key biomarker and therapeutic target, thereby enhancing our understanding of sepsis management and paving the way for the future.^[42,43]^

The negative correlation between *B3GALT6* and sepsis is novel. *B3GALT6* encodes a glycosyltransferase (GAG) that catalyzes the transfer of galactose from UDP-galactose to a substrate containing the terminal β-linked galactose moiety, an enzyme that is required for glycosaminoglycan synthesis.^[44]^ GAGs (heparan-, chondroitin/dermatan-, and hyaluronan chains) are structural and functional constituents of the endothelial glycocalyx and of cell-surface/ECM proteoglycans that regulate leukocyte adhesion, complement activation, barrier permeability, and growth-factor/cytokine presentation. Disruption or enzymatic degradation of the glycocalyx is a central pathophysiological event in sepsis and contributes to microvascular leakage, inflammation, and organ dysfunction.^[45]^ This is a critical process for extracellular matrix integrity and signaling of immune cells.^[46]^ Clinical and experimental sepsis literature highlights GAG/glycocalyx degradation as a mechanistic driver of endothelial dysfunction; circulating GAG fragments correlate with disease severity, and interventions that preserve or restore glycocalyx components reduce vascular permeability and inflammation in preclinical/early clinical studies. Together, these observations provide a plausible mechanistic route by which genetically determined reductions in B3GALT6 activity (or secondary downregulation in sepsis) could worsen endothelial injury and inflammatory dysregulation.^[47]^ Its downregulation in sepsis may impair leukocyte adhesion and endothelial barrier function, exacerbating organ dysfunction.^[48]^ The bioinformatics technology identified 9 shared DEGs across sepsis datasets, enriched in pathways such as endocytosis, glycosaminoglycan biosynthesis – chondroitin sulfate/dermatan sulfate, and necroptosis. These pathways are mechanistically linked to sepsis pathogenesis: Dying cells (including necrotic apoptotic cells) can release a large number of danger signals, thus promoting inflammation and sepsis deterioration,^[49]^ while dysregulated endocytosis in immune cells compromises pathogen clearance.^[50]^ The enrichment of ESCRT complex-related terms (e.g., multivesicular body assembly) aligns with recent evidence implicating extracellular vesicle-mediated inflammation in sepsis.^[51]^ Previous studies demonstrated that genetically elevated IL-6 and TNF-α levels increase sepsis risk and mortality, supporting the central role of inflammatory mediators in sepsis pathogenesis.^[38]^ Unlike those cytokine-focused MR analyses, which mainly identify downstream inflammatory biomarkers, our study integrates pQTL, eQTL, and transcriptomic validation and identifies B3GALT6 as a druggable upstream regulator with mechanistic links to endothelial integrity and host–pathogen interactions. This highlights *B3GALT6* as a biologically distinct target compared to previously reported cytokine markers.

This study advances beyond previous work, who identified PSMA4 (proteasome-mediated inflammation) as a sepsis target using eQTL-only MR.^[12]^ In contrast, our integration of pQTL and eQTL strengthens causal links between genetic variation, protein abundance, and disease – a critical step for druggability validation. Additionally, we provide functional characterization (pathway enrichment, PPI networks) and diagnostic potential assessment, addressing gaps in earlier research.

Limitations include reliance on European ancestry datasets, which may limit generalizability. Functional validation in in vitro/in vivo models is needed to confirm *B3GALT6*’s protective mechanisms, and reverse causation (e.g., sepsis-induced epigenetic changes) cannot be fully excluded. Future studies should explore *B3GALT6* interactions with other targets (e.g., *TIMP4*) and evaluate its therapeutic potential in diverse populations.

In conclusion, this study identifies *B3GALT6* as a genetically validated, functionally relevant target for sepsis, with diagnostic and therapeutic implications. By integrating multi-omics data, we extend the sepsis target discovery paradigm, highlighting the value of combining MR with pQTL/eQTL and bioinformatics to bridge genetic association and clinical translation.

## 5. Conclusion

By integrating multi-omics data, this study identifies *CRP* and *B3GALT6* as key causal mediators of sepsis, with opposing effects on disease risk. The protective role of *B3GALT6*, supported by robust MR and bioinformatics evidence, positions it as a novel therapeutic candidate. These findings underscore the value of leveraging genetic instruments to disentangle complex disease mechanisms and accelerate drug discovery in critical care.

## Acknowledgments

We are willing to show our respect to the participants and investigators of the FinnGen study, eQTLGen study, Zheng et al, and Ferkingstad et al. And we also acknowledge GEO, HPA for providing their platforms and contributors for uploading their meaningful datasets, and all the patients who gave consent to disclose their medical records.

## Author contributions

**Conceptualization:** Enkui Lu, Weidong Tang.

**Data curation:** Yanan Zhang, Xuebo Shao.

**Funding acquisition:** Enkui Lu.

**Methodology:** Xuebo Shao, Junnan Chen.

**Resources:** Enkui Lu.

**Software:** Junnan Chen.

**Supervision:** Enkui Lu, Mengping Jiang, Weidong Tang.

**Writing – original draft:** Yanan Zhang.

**Writing – review & editing:** Enkui Lu, Mengping Jiang.
